# Citrate Promotes Nitric Oxide Production during Human Sperm Capacitation

**DOI:** 10.3390/antiox13080885

**Published:** 2024-07-23

**Authors:** Diego Loggia, Cristian O’Flaherty

**Affiliations:** 1 Department of Pharmacology and Therapeutics, Faculty of Medicine and Health Sciences, McGill University, Montreal, QC H3G 1Y6, Canada; 2 Department of Surgery, Urology Division, Faculty of Medicine and Health Sciences, McGill University, Montreal, QC H4A 3J1, Canada; 3 The Research Institute, McGill University Health Centre, Montreal, QC H4A 3J1, Canada; 4 Department of Anatomy and Cell Biology, Faculty of Medicine and Health Sciences, McGill University, Montreal, QC H3A 0C7, Canada

**Keywords:** sperm capacitation, citrate, reactive oxygen species (ROS), nitric oxide (NO^●^), male fertility

## Abstract

Sperm capacitation is a complex process essential for the spermatozoon to recognize and fertilize the oocyte. For capacitation to occur, human spermatozoa require low levels of reactive oxygen species (ROS), increased protein tyrosine phosphorylation, and sufficient levels of energy metabolites such as citrate. Human spermatozoa are exposed to high concentrations of citrate from the seminal plasma, yet the role of citrate in sperm capacitation is largely unknown. We report that citrate can support capacitation in human spermatozoa incubated with no other energy metabolites in the capacitation medium. Reduced capacitation levels were observed in spermatozoa incubated with inhibitors of mitochondrial citrate transporter (CIC), cytosolic ATP-citrate lyase (ACLY), malic enzyme (ME), and nitric oxide synthase (NOS). The role of citrate metabolism in ROS production was further elucidated as citrate increased NO^●^ production in capacitated spermatozoa, whereas inhibition of ACLY reduced NO^●^ production. This research characterizes a novel metabolic pathway for citrate to produce NO^●^ in the process of human sperm capacitation.

## 1. Introduction

Human infertility is a growing global concern, as approximately one in six couples currently suffer from infertility worldwide [1,2]. It has been reported that nearly half of infertility cases are caused by male-related factors, including varicocele, hypogonadism, or urogenital infections [3,4]. However, in almost 34% of male infertility cases, the cause of infertility cannot be explained, presenting an unmet need to characterize the mechanisms underlying male infertility [5].

To recognize and fertilize the oocyte, mammalian spermatozoa must undergo a series of time-dependent biochemical and morphological changes during the process of capacitation. Capacitation-associated events include an increase in intracellular pH and calcium, the efflux of cholesterol from the sperm plasma membrane to increase its fluidity, the generation of low and controlled amounts of reactive oxygen species (ROS), and activation of several protein kinases that promote phosphorylation of proteins in serine, threonine, and tyrosine residues to activate several ion channels and enzymes. The changes that occur during capacitation allow the spermatozoon to acquire hyperactive movement and the ability to respond to progesterone and glycoproteins of the zona pellucida (ZP) to undergo the acrosome reaction, the exocytotic event that allows the spermatozoon to dissolve the zona pellucida to reach the oocyte and fertilize it [6,7,8,9,10,11,12,13]. In humans, sperm capacitation takes four to ten hours, requires a temperature of 37 °C, and occurs in the female oviduct [14,15]. However, capacitation can be reproduced in vitro by incubating spermatozoa in specialized media supplemented with well-established capacitation inducers such as fetal cord serum ultrafiltrate (FCSu) or bovine serum albumin (BSA) with bicarbonate [16,17].

Sperm capacitation requires the low and controlled production of ROS, such as the superoxide anion (O_2_^●−^), hydrogen peroxide (H_2_O_2_), and nitric oxide (NO^●^) [18,19]. These ROS trigger a series of timely and finely regulated signaling pathways including cAMP/PKA, PKC, PI3K/AKT, ERK, and PTK, resulting in tyrosine phosphorylation events necessary to promote capacitation [8,9,10,11]. Extracellular O_2_^●−^ production occurs during the first 30 min of initiated capacitation, whereas NO^●^ is produced throughout the capacitation process [17,20,21]. To produce NO^●^, sperm nitric oxide synthases (NOS; neuronal, endothelial, and inducible) use NADPH and molecular oxygen to catalyze the oxidation of L-arginine to NO^●^ and L-citrulline. Given that NOS enzymes are distributed throughout the spermatozoon, and the permeability of NO^●^ allows it to act both extracellularly and intracellularly, the activity of NO^●^ generated during capacitation may not be limited to one cellular compartment [22,23]. Though it has been shown that the generation of this NO^●^ is essential for capacitation, the mechanism of its regulation during the process remains largely unclear.

Citrate is an organic carboxylic acid whose primary function is to supply energy to cells through the citric acid cycle within the mitochondrion. Human spermatozoa are exposed to high levels of citrate from the seminal plasma, originating primarily from the prostate [24]. It has been reported that the concentration of citrate in the seminal plasma of fertile men ranges from 5–50 mM, whereas levels of citrate are considerably lower in the seminal plasma of some infertile men [24,25,26,27,28]. Despite these high levels of citrate that fertile human spermatozoa are exposed to, few studies have investigated the relationship between citrate metabolism and male fertility, and the role of citrate in redox signaling during human sperm capacitation has yet to be elucidated.

Citrate is typically associated with the citric acid cycle and mitochondrial respiration, producing ATP which is required by the spermatozoon to sustain cell viability, flagellar motility, and phosphorylation events in various signaling pathways [29,30]. However, the metabolism of cytosolic citrate can also play an important role in the regulation of key cellular processes. Extracellular citrate can be imported into the cytosol via sodium-coupled solute carrier proteins (NaCT), and mitochondrial citrate can be exported to the cytosol via the mitochondrial citrate transport protein (CIC/SLC25A1). Cytosolic citrate can then be converted by the ATP- and Coenzyme A-dependent enzyme known as ATP-citrate lyase (ACLY) to yield acetyl-CoA and oxaloacetate, which participate in unique metabolic pathways. Acetyl-CoA may be used in various processes, including de novo fatty acid synthesis and protein acetylation. Meanwhile, oxaloacetate can be converted by malate dehydrogenase (MDH) to produce malate and NAD+, using cytosolic NADH as a cofactor. The malic enzyme (ME) can then convert malate and NADP+ to generate pyruvate and NADPH. The pyruvate produced in this process can then re-enter the mitochondrion to provide intermediates of the Krebs cycle and continue cellular respiration, and the NADPH can serve many functions within the spermatozoon. For instance, NADPH can be used in the biosynthesis of fatty acids for long-term energy storage or in the supply of reducing equivalents for the thioredoxin/thioreductase system involved in the antioxidant response of spermatozoa [31,32]. NADPH is also known to play a role in NO^●^ production as it serves as a cofactor for NOS activity, providing a potential connection between citrate metabolism and NO^●^ generation [33]. Indeed, CIC and ACLY are involved in NO^●^ production in macrophages during inflammation [34,35,36]. However, the link between the NADPH produced from cytosolic citrate metabolism and intracellular NO^●^ production in capacitating spermatozoa has never been evaluated.

In this study, we hypothesized that cytosolic citrate supports human sperm capacitation by generating NO^●^. Our objectives were to determine the participation of mitochondrial CIC, ACLY, ME, and NOS in the process of NO^●^ production during human sperm capacitation.

## 2. Materials and Methods

### 2.1. Materials

Percoll was purchased from GE Healthcare (Montreal, QC, Canada). Nitrocellulose membranes (pore size, 0.22 mm) were purchased from Osmonics, Inc. (Westborough, MA, USA). The primary antibodies, anti-phosphotyrosine and anti-phospho-ATP-citrate lyase, were supplied by Cell Signaling Technology, Inc. (Beverly, MA, USA). The peroxidase-conjugated secondary antibodies, goat anti-mouse IgG and donkey anti-rabbit IgG, were supplied by Jackson ImmunoResearch Laboratories, Inc. (West Grove, PA, USA). Pierce^TM^ ECL Western blotting substrate was purchased from ThermoScientific (Rockford, IL, USA). The birthing center of the McGill University Health Center provided fetal cord serum. Trisodium citrate dihydrate was purchased from BioShop (Burlington, ON, Canada). Mitochondrial citrate transport protein inhibitor, SB204990, and bromopyruvic acid were purchased from MilliporeSigma (Burlington, MA, USA). L-N^G^-Nitro arginine methyl ester (L-NAME) was purchased from Cayman Chemicals (Ann Arbor, MI, USA). Pisum sativum agglutinin conjugated to FITC (PSA-FITC) was purchased from Sigma-Aldrich Chemical Co. Diaminofluorescein-2 diacetate (DAF-2DA) was supplied by Calbiochem (San Diego, CA, USA). The other chemicals used were of reagent grade or higher.

### 2.2. Human Sperm Preparation

Semen samples were obtained from 20 fertile volunteers between the ages of 21 and 30 years old by masturbation after 72 h of sexual abstinence. All donors were recruited at the McGill University Health Centre with informed consent and were non-smokers and of good health with no known illnesses. The exclusion criteria were the presence of abnormal semen parameters (low sperm count (>200 × 10^6^ spermatozoa/mL), motility (≥70% progressive motility), and normal spermatozoa (≥32%) according to World Health Organization (WHO) guidelines on semen evaluation [37], and the inability of spermatozoa to undergo in vitro capacitation and acrosome reaction. Samples were then incubated at 37 °C for 30 min to allow liquefaction followed by centrifugation at 2300× *g* for 30 min at room temperature over a four-layer Percoll gradient (95%, 60%, 40%, and 20% Percoll bottom-to-top, made with isotonic HEPES buffered saline (HBS) and deionized water). Highly motile spermatozoa from the 95% fraction and the 65–95% interface were recovered and diluted to 150 × 10^6^ or 250 × 10^6^ cells/mL in Biggers, Whitten, and Whittingham medium (BWW-REG; 91.5 mM NaCl, 4.6 mM KCl, 1.7 mM CaCl_2_, 1.2 mM KH_2_PO_4_, 1.2 mM MgSO_4_, 5.6 mM D-glucose, 0.25 mM sodium pyruvate, 21.6 mM sodium lactate, and 20 mM HEPES, pH 7.95) [38]. In experiments that required the absence of energy substrates, spermatozoa were instead prepared in BWW medium with no energy substrates (BWW-NES), omitting the addition of sodium pyruvate, sodium lactate, and D-glucose. The prepared sperm samples were diluted to 30 × 10^6^ or 50 × 10^6^ cells per mL (either in BWW-REG or BWW-NES) and supplemented with 10% fetal cord serum ultrafiltrate (FCSu), a known inducer of capacitation in human spermatozoa [39]. Each sample was incubated for 3.5 h at 37 °C in the presence and absence of the following compounds: trisodium citrate (5, 10, 15, and 20 mM), mitochondrial citrate transport inhibitor (0.5 mM), the ACLY inhibitor SB204990 (30 μM), the ME inhibitor bromopyruvic acid (100 μM), and the NOS inhibitor L-NAME (1 mM). Concentrations of citrate and each of the inhibitors used were considered based on the reported concentrations of citrate in the seminal plasma of fertile men, as well as IC_50_ values.

### 2.3. SDS-PAGE and Immunoblotting

Each prepared human sperm sample was supplemented with electrophoresis sample buffer containing 100 mM dithiothreitol (DTT) and phosphatase inhibitors (0.1 mM sodium vanadate, 20 mM glycerol phosphate, and 5 mM sodium fluoride). Samples were then heated at 97 °C for 5 min and centrifuged at 21,000× *g* for 5 min at room temperature. The supernatant of each sample was loaded into a 10% polyacrylamide gel, electrophoresed, and electrotransferred onto a nitrocellulose membrane. The membranes were blocked for 1 h with 5% skim milk in Tris-buffered saline with Tween 20 (TTBS 1X), followed by overnight incubation at 4 °C with either anti-phosphotyrosine or anti-phospho-ATP-citrate lyase primary antibodies (1:10,000 and 1:500, respectively) in antibody buffer (TBS 1X, 0.1% Tween 20, 25 mg/mL BSA, and deionized water). Following primary antibody incubation, the membranes were washed 5 times for 10 min in TTBS 1X and incubated for 1 h in either goat anti-mouse or donkey anti-rabbit secondary antibodies (diluted at 1:2500 in TTBS 1X) at room temperature. Once the secondary antibody was removed, the membranes were washed 5 times for 10 min in TTBS 1X and soaked in ECL solution (luminol enhancer and peroxide) for 5 min. Imaging was carried out using the Amersham Imager 600 supplied by GE Healthcare (Montreal, QC, Canada). The relative intensity of each protein band was determined using Image J software version 2.1.0/1.53c (National Institutes of Health, Bethesda, MD, USA), standardized to the corresponding band on the silver-stained membrane, and normalized to the respective control.

### 2.4. Silver Staining

Following SDS-PAGE and immunoblotting, the nitrocellulose membranes were washed 3 times for 10 min in deionized water and incubated in silver stain solution (2% *w*/*v* trisodium citrate, 0.8% *w*/*v* FeSO_4_, and 0.2% *w*/*v* AgNO_3_ in deionized water) [40]. Once a maximum signal was obtained, the membrane was washed with deionized water. Two to three drops of Farmer’s reducer (0.05% sodium carbonate, 0.15% potassium hexacyanoferrate, and 0.3% thiosulfate) was then added to the water to enhance staining, followed by membrane imaging.

### 2.5. Sperm Motility and Viability Analysis

Sperm samples were loaded onto a Makler chamber, and sperm motility was determined using the HT-IVOS II CASA system set at 37 °C (Hamilton Thorne, Beverly, MA, USA). The total and progressive motility values of at least 200 spermatozoa were saved for analysis.

To determine sperm viability, the prepared sperm samples were incubated in hypo-osmotic swelling solution (75 mM fructose and 25 mM sodium citrate dihydrate) for 30 min at 37 °C. The samples were then loaded onto a Superfrost microscope slide provided by Fisher Scientific (Ottawa, ON, Canada) and covered with a cover slip. A minimum of 200 spermatozoa were counted at 20X magnification using the HT-IVOS II CASA microscope, and the percentage of viable spermatozoa was determined based on the WHO guidelines [37].

### 2.6. Nitric Oxide Quantification

Highly motile Percoll-selected preparations of human spermatozoa at 250 × 10^6^ cells per mL were incubated with 10 μM diaminofluorescein-2 diacetate (DAF-2 DA, a sensitive and specific fluorescent probe for intracellular NO^●^) for 30 min at 37 °C [21]. The treated samples were then diluted to 50 × 10^6^ cells per mL and incubated for 3.5 h at 37 °C with or without 10% FCSu, citrate, and/or 30 μM ACLY inhibitor SB204990. Each sample was centrifuged for 5 min at 600× *g*, and the pellet was resuspended in HBS 1X containing 2% paraformaldehyde. A 10 μL aliquot was pipetted onto a Superfrost slide, and a drop of ProLong Antifade with 4′,6-diamidino-2-phenylindole (DAPI) was added. The samples were then smeared across the microscope slide and covered with a cover slip. Fluorescence intensity for at least 200 spermatozoa was analyzed using a Zeiss Axiophot microscope with a 450–490 nm bandpass excitation filter, and levels of NO^●^ were determined by obtaining the average corrected total cell fluorescence (CTCF) using Image J software.

### 2.7. Citrate Quantification

Citrate levels were measured by spectrofluorometry using an enzyme-based kit (ab83396) from Abcam (Toronto, ON, Canada). Samples of Percoll-selected spermatozoa were incubated in BWW-NES at 2 × 10^6^ cells per tube, with or without FCSu and 0.5 mM CIC inhibitor, either before or after an incubation period at 37 °C for 3.5 h. Each sample was deproteinized using 4 M perchloric acid (PCA) and 2 M potassium hydroxide (KOH), and fluorescence was measured at Ex/Em 535/587 nm using a TECAN M200 Pro Multimode plate reader (Männedorf, ZH, Switzerland). Since 10% FCSu contained 36 μM ± 2.1 SEM citrate, the fluorescence of non-capacitated and FCSu-containing samples were normalized to their respective controls (0 h without the CIC inhibitor) and expressed in arbitrary units (a.u.).

### 2.8. Assessment of Sperm Acrosome Reaction

Sperm samples were capacitated with FCSu at 37 °C for 3.5 h in BWW-REG following treatment with 0.5 mM CIC inhibitor, 30 μM SB204990 (ACLY inhibitor), or 100 μM bromopyruvic acid (ME inhibitor) in the presence and absence of citrate. Samples were then centrifuged for 5 min at 600× *g*, and the pellet was resuspended in fresh BWW medium containing 10 μM progesterone upon incubation at 37 °C for 30 min to induce the acrosome reaction [41]. Each sample was centrifuged for 5 min at 600× *g*, and the pellet was fixed in 95% ethanol. Samples were smeared on a Superfrost microscope slide, air dried, and incubated 30 μg/mL PSA-FITC at 37 °C for 5 min. Slides were washed and covered with a coverslip, and the percentage of reacted acrosomes among 200 spermatozoa was determined using a Leica DMI6000 microscope (Leica Microsystems, Wetzlar, Germany).

### 2.9. Statistical Analysis

All results are presented as mean ± standard error. The normal data distribution was confirmed using the Shapiro–Wilk test. The homogeneity of variances was determined using the Bartlett test. The ANOVA test was used to determine whether there were statistical differences between groups, and Tukey’s test was used to assess specific post hoc comparisons among groups. The *p* ≤ 0.05 value was regarded as statistically significant.

## 3. Results

### 3.1. Citrate Supports Human Sperm Capacitation

**Figure 1:**
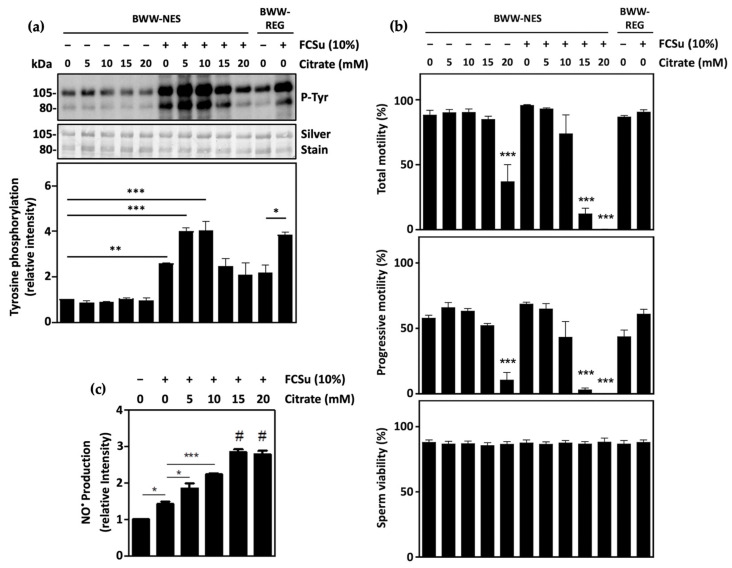
Citrate supports human sperm capacitation. (**a**) Human spermatozoa incubated with 5 and 10 mM citrate in BWW-NES (containing no energy substrates) supported tyrosine phosphorylation (P-Tyr) when FCSu was present, at levels comparable to spermatozoa incubated with FCSu in regular BWW (BWW REG containing pyruvate, lactate, and glucose). (**b**) Incubation of capacitating spermatozoa with 5 and 10 mM citrate has no effect on total sperm motility, progressive motility, or sperm viability. Incubation with 15 and 20 mM citrate impairs total and progressive motility, with no impact on sperm viability. (**c**) NO^●^ quantification using DAF-2DA reveals increased intracellular NO^●^ production with FCSu, with a further increase in spermatozoa supplemented with 5, 10, 15, and 20 mM citrate. The results were obtained with sperm samples from different healthy donors. Relative intensities and percent values are expressed as mean ± SEM (* *p* ≤ 0.05, ** *p* ≤ 0.01, *** *p* ≤ 0.005, # means higher than other samples, ANOVA and Tukey’s test, *n* = 4).

Sperm capacitation was determined by quantifying protein tyrosine phosphorylation levels in spermatozoa incubated in BWW-NES (lacking energy substrates pyruvate, lactate, and glucose). The addition of the capacitation inducer FCSu in these samples promoted an increase in tyrosine phosphorylation, which was further increased when spermatozoa were supplemented with 5 and 10 mM citrate (Figure 1a). In samples incubated with both 5 and 10 mM citrate, levels of capacitation were comparable to spermatozoa incubated with FCSu in BWW-REG containing pyruvate, lactate, and glucose. Importantly, the total motility, progressive motility, and viability of spermatozoa incubated with 5 and 10 mM citrate with or without FCSu were not significantly impaired, indicating that these citrate concentrations have no effect on overall sperm health (Figure 1b). These results indicate that citrate alone cannot promote sperm capacitation but is sufficient to support it when spermatozoa are incubated with FCSu.

It should be noted, however, that spermatozoa incubated with higher concentrations of citrate (15 and 20 mM) in BWW-NES exhibited decreased tyrosine phosphorylation, at levels comparable to spermatozoa incubated in FCSu alone. The supplementation of spermatozoa with these high concentrations of citrate also impaired total and progressive motility, but not viability. In the absence of FCSu, the incubation of spermatozoa with 20 mM citrate significantly reduced total and progressive motility; in the presence of FCSu, supplementation with both 15 and 20 mM citrate impaired motility.

In addition, following incubation of samples for 3.5 h with FCSu, levels of intracellular NO^●^ were found to be increased in spermatozoa incubated with citrate in a dose-dependent manner (Figure 1c). This result highlights that citrate may be supporting capacitation through the production of low levels of NO^●^, yet excessive citrate may promote oxidative stress, leading to the impairment of capacitation.

### 3.2. Cytosolic Citrate Is Consumed during Human Sperm Capacitation In Vitro

**Figure 2:**
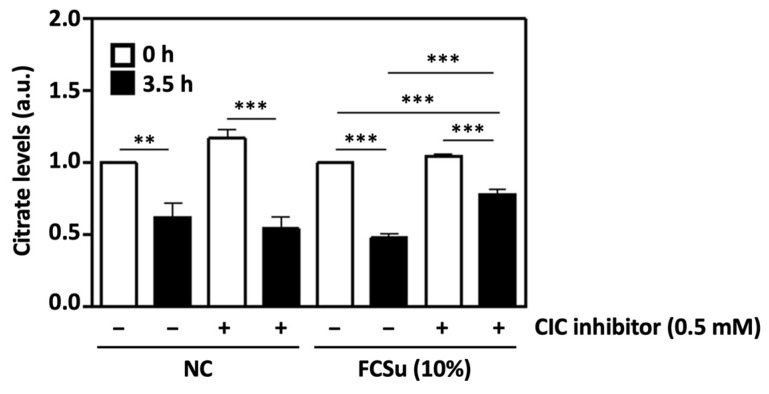
Cytosolic citrate is consumed in the process of human sperm capacitation. Spermatozoa incubated in non-capacitating conditions (NC) for 3.5 h at 37 °C in BWW-NES exhibit decreased citrate levels compared to samples at 0 h. In samples containing the capacitation inducer FCSu, the presence of 0.5 mM CIC inhibitor leads to decreased citrate consumption after 3.5 h when compared to samples incubated in FCSu alone. The results were obtained with sperm samples from different healthy donors. Citrate levels are expressed in arbitrary units (a.u.) ± SEM and normalized to the NC or FCSu controls at 0 h, representing two distinct groups (** *p* ≤ 0.01, *** *p* ≤ 0.001, ANOVA and Tukey’s test, *n* = 6).

Next, citrate levels were quantified in spermatozoa incubated in BWW-NES with 0.5 mM of a competitive inhibitor of the mitochondrial citrate transporter (CIC) and FCSu as a capacitation inducer. After 3.5 h of incubation at 37 °C, levels of citrate were significantly decreased in all samples (Figure 2). In non-capacitated samples (lacking FCSu), the presence of the CIC inhibitor did not affect levels of citrate at 0 or 3.5 h of incubation. However, in samples containing FCSu after 3.5 h, higher citrate levels were observed in spermatozoa capacitated in the presence of the CIC inhibitor when compared to samples capacitated in FCSu alone, indicating decreased citrate consumption. The concentration of citrate in 10% FCSu was found to be 36 μM ± 2.1 SEM.

### 3.3. Mitochondrial Citrate Transport Supports Human Sperm Capacitation

**Figure 3:**
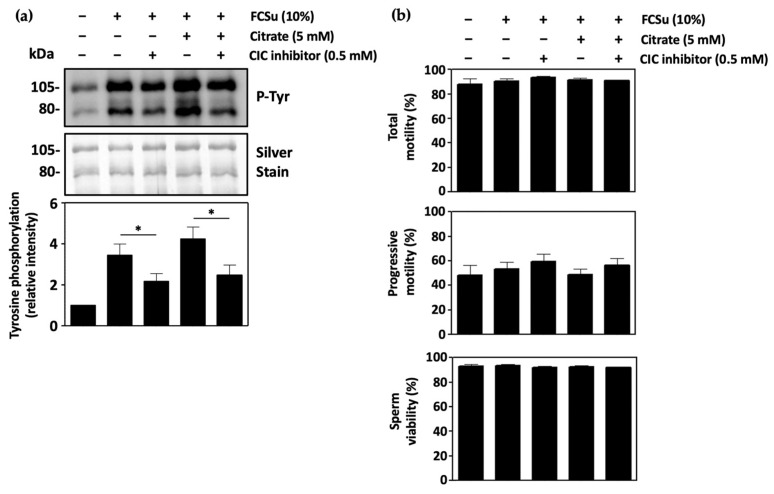
Mitochondrial citrate transport supports human sperm capacitation. (**a**) Spermatozoa capacitated with FCSu in BWW-REG in the presence of 0.5 mM of a competitive inhibitor of the mitochondrial citrate transporter (CIC) exhibit decreased tyrosine phosphorylation (P-Tyr) in the presence and absence of 5 mM citrate, indicating an impairment of capacitation. (**b**) Inhibition of mitochondrial CIC does not affect total sperm motility, progressive motility, or sperm viability. The results were obtained with sperm samples from different healthy donors. Relative intensities and percent values are expressed as mean ± SEM (* *p* ≤ 0.05, ANOVA and Tukey’s test, *n* = 4).

The participation of mitochondrial citrate transport in sperm capacitation was then established in human spermatozoa capacitating in regular BWW medium (BWW-REG) with 0.5 mM CIC inhibitor. The presence of CIC inhibitor in the medium prevented FCSu-induced capacitation, as determined by the low tyrosine phosphorylation levels in treated spermatozoa both in the presence and absence of citrate (Figure 3a). This effect was consistent in spermatozoa capacitated in BWW-NES and in samples incubated in BWW-REG with 3 mg/mL BSA and 25 mM bicarbonate (another known capacitation inducer system) (Supplementary Figures S1 and S2, respectively). Importantly, total sperm motility, progressive motility, and sperm viability were all unaffected by both citrate and the mitochondrial CIC inhibitor, demonstrating that any effect of reduced tyrosine phosphorylation was not due to any toxic effects of the CIC inhibitor (Figure 3b).

### 3.4. ATP-Citrate Lyase Supports Human Sperm Capacitation

**Figure 4:**
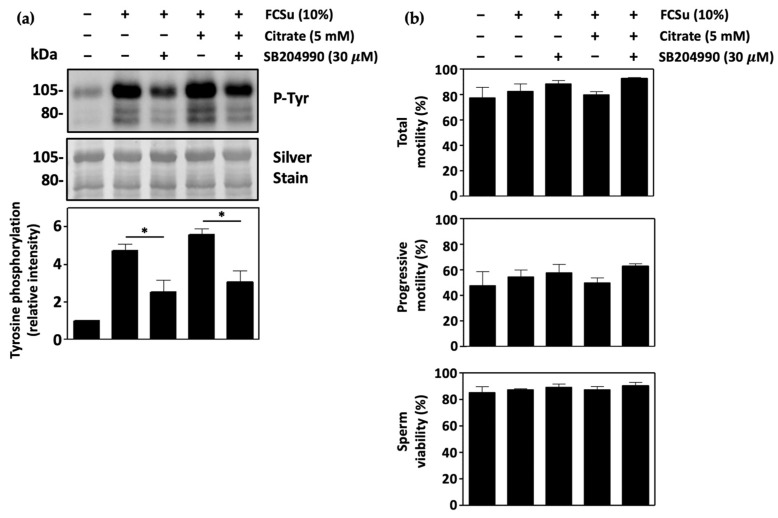
ATP-citrate lyase supports human sperm capacitation. (**a**) Spermatozoa capacitating in BWW-REG with 30 μM SB204990, an inhibitor of ATP-citrate lyase (ACLY), show decreased tyrosine phosphorylation in the presence and absence of 5 mM citrate. (**b**) Incubation of spermatozoa with 30 μM SB204990 or citrate has no effect on total sperm motility, progressive motility, or sperm viability. The results were obtained with sperm samples from different healthy donors. Relative intensities and percent values are expressed as mean ± SEM (* *p* ≤ 0.05, ANOVA and Tukey’s test, *n* = 4).

We then investigated the role of ATP-citrate lyase (ACLY) in sperm capacitation by determining tyrosine phosphorylation levels in spermatozoa capacitating in BWW-REG with SB204990, an inhibitor of ACLY. Tyrosine phosphorylation levels were decreased in spermatozoa treated with 30 μM SB204990 in the presence and absence of citrate (Figure 4a). The inhibition of sperm ACLY also decreased levels of tyrosine phosphorylation in BWW-NES and in BWW-REG with BSA and bicarbonate as an inducer of capacitation (Supplementary Figures S1 and S2, respectively). No effect on total sperm motility, progressive motility, or sperm viability was observed, demonstrating no effect on overall sperm health (Figure 4b).

### 3.5. ATP-Citrate Lyase Is Activated during Sperm Capacitation

**Figure 5:**
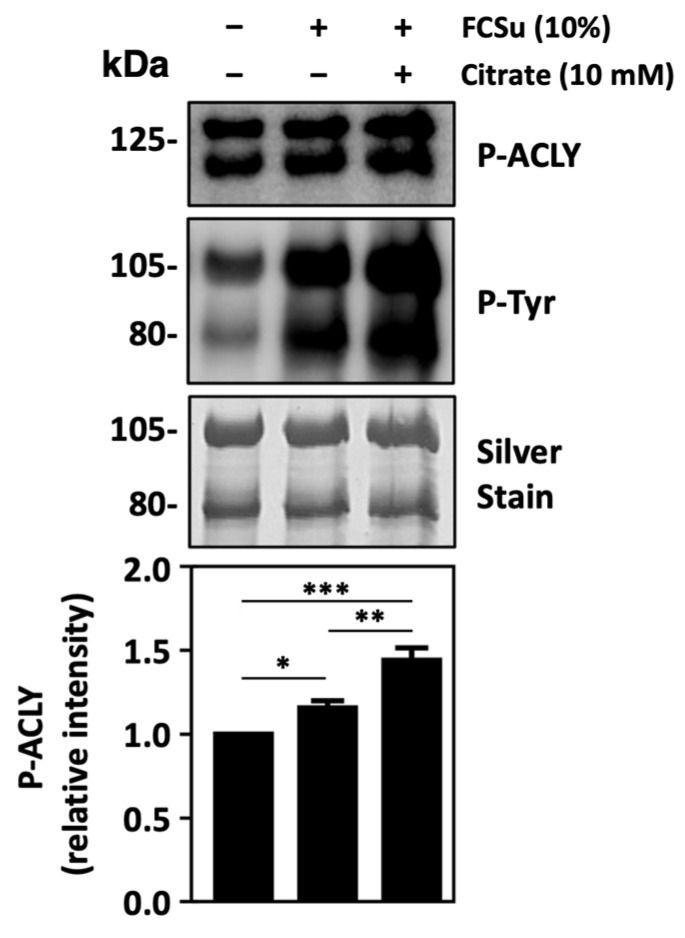
ATP-citrate lyase is activated during human sperm capacitation. Phospho-ATP-citrate lyase (P-ACLY) levels reveal increased ACLY activation in spermatozoa incubated in BWW-REG with the capacitation inducer FCSu, which is further increased in the presence of 10 mM citrate. Levels of P-ACLY are consistent with tyrosine phosphorylation patterns in the same samples. The results were obtained with sperm samples from different healthy donors. Relative intensities are expressed as mean ± SEM (* *p* ≤ 0.05, ** *p* ≤ 0.01, *** *p* ≤ 0.005 ANOVA and Tukey’s test, *n* = 6).

We followed by examining whether ACLY activation occurs during sperm capacitation by determining levels of phosphorylated ACLY (P-ACLY). P-ACLY levels were found to be increased in spermatozoa incubated in BWW-REG supplemented with the capacitation inducer FCSu and further increased when citrate was present in the capacitation media (Figure 5). We also observed increased ACLY activation in spermatozoa capacitated with FCSu and citrate in BWW-NES (Supplementary Figure S3). These results aligned well with the patterns of tyrosine phosphorylation that were observed, where citrate increased levels of FCSu-induced capacitation.

### 3.6. Malic Enzyme Supports Human Sperm Capacitation

**Figure 6:**
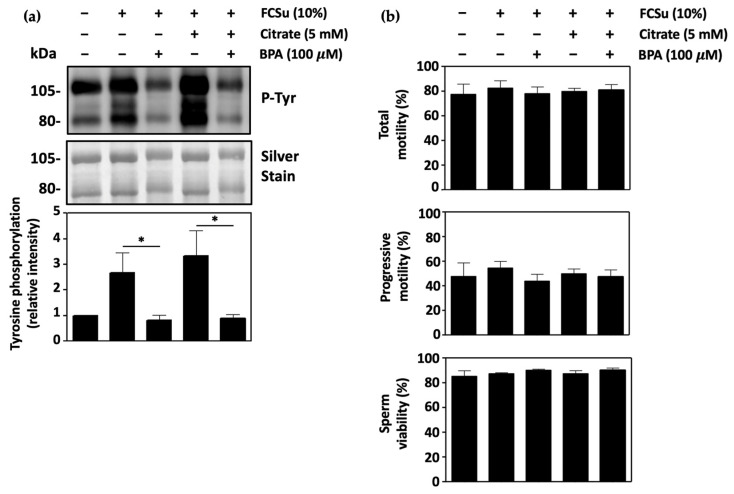
Malic enzyme supports human sperm capacitation. (**a**) Spermatozoa capacitated in BWW-REG with FCSu in the presence of 100 μM bromopyruvic acid (BPA), an inhibitor of the malic enzyme (ME), show decreased tyrosine phosphorylation in the presence and absence of 5 mM citrate. (**b**) Incubation of spermatozoa with 100 μM BPA or citrate has no effect on total motility, progressive motility, or viability of capacitating spermatozoa. The results were obtained with sperm samples from different healthy donors. Relative intensities and percent values are expressed as mean ± SEM (* *p* ≤ 0.05, ANOVA and Tukey’s test, *n* = 4).

The next step was to determine the importance of the malic enzyme (ME) in sperm capacitation. Here, capacitating spermatozoa were incubated in BWW-REG with 100 μM bromopyruvic acid (BPA), a non-competitive inhibitor of ME, in the presence and absence of citrate. In each condition, the incubation of capacitating spermatozoa with BPA led to a significant decrease in capacitation (Figure 6a). The same effect was observed in spermatozoa incubated in BWW-NES, and in samples incubated in BWW-REG with BSA and bicarbonate as an inducer of capacitation (Supplementary Figures S1 and S2, respectively). The presence of BPA did not affect total sperm motility, progressive motility, or sperm viability (Figure 6b). Thus, the conversion of malate to pyruvate and NADPH was found to play an important role in the process of capacitation.

### 3.7. Citrate Metabolism Supports the Ability of Spermatozoa to Undergo Acrosome Reaction

**Figure 7:**
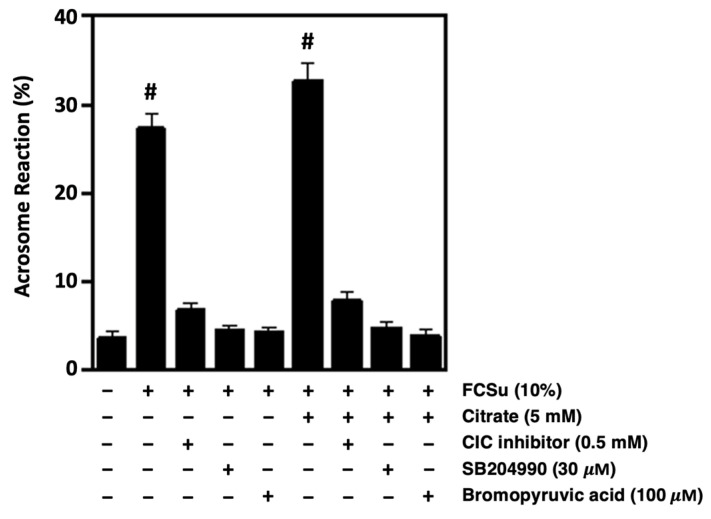
Citrate metabolism supports the ability of spermatozoa to undergo the acrosome reaction. Incubation of spermatozoa in BWW-REG with 0.5 mM CIC inhibitor, 30 μM SB204990 (ACLY inhibitor), or 100 μM bromopyruvic acid (ME inhibitor) decreases the percentage of progesterone-induced acrosome reaction in the presence and absence of 5 mM citrate. The results were obtained with sperm samples from different healthy donors. Percent values are expressed as mean ± SEM (# means higher than other samples, ANOVA and Tukey’s test, *n* = 4).

To validate whether CIC, ACLY, and ME are involved in sperm capacitation, the ability of spermatozoa to undergo the acrosome reaction was assessed, as only capacitated spermatozoa may undergo this process. Spermatozoa capacitated with FCSu in BWW-REG were incubated with the CIC inhibitor, SB204990 (ACLY inhibitor), or bromopyruvic acid (ME inhibitor) in the presence and absence of citrate. Following the 3.5-h incubation period, only samples treated with FCSu or FCSu with 5 mM citrate could undergo the progesterone-induced acrosome reaction (Figure 7). Notably, samples incubated with each of the respective inhibitors exhibited significantly lower levels of the acrosome reaction and were comparable to that observed in the non-capacitated control. These results were consistent with the tyrosine phosphorylation described above and confirm the need for citrate metabolism in human sperm capacitation.

### 3.8. Nitric Oxide Production Is Involved in Citrate-Mediated Capacitation

**Figure 8:**
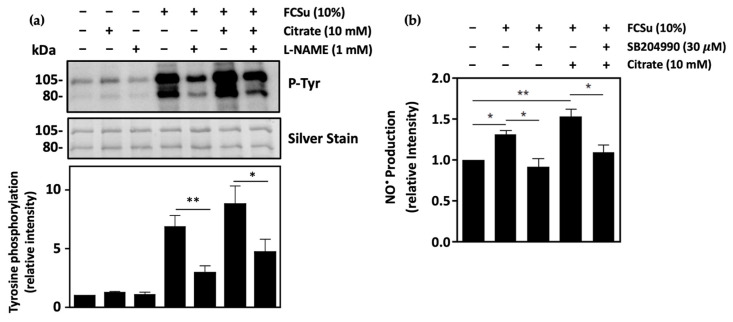
Nitric oxide production is required for citrate-mediated capacitation. (**a**) Human spermatozoa incubated in BWW-REG with 1 mM L-N^G^-Nitro arginine methyl ester (L-NAME, an inhibitor of nitric oxide synthase) show decreased capacitation in the presence and absence of 10 mM citrate, with FCSu as an inducer of capacitation. (**b**) NO^●^ quantification using DAF-2DA reveals decreased NO^●^ production in capacitating spermatozoa incubated in BWW-REG with the ACLY inhibitor SB204990 but increased NO^●^ production in spermatozoa incubated with citrate. The results were obtained with sperm samples from different healthy donors. Relative intensities and percent values are expressed as mean ± SEM (* *p* < 0.05, ** *p* ≤ 0.01, ANOVA and Tukey’s test, *n* = 4).

To elucidate whether NO^●^ synthesis occurs in citrate-mediated capacitation, spermatozoa capacitated with FCSu were supplemented with 1 mM L-N^G^-Nitro arginine methyl ester (L-NAME), with or without 10 mM citrate. L-NAME is a competitive inhibitor of NOS previously reported to decrease capacitation in mammalian spermatozoa [20,42]. Here, the incubation of spermatozoa in L-NAME resulted in a significant decrease in tyrosine phosphorylation in the presence and absence of citrate, validating the need for NO^●^ in sperm capacitation (Figure 8a). The same effect of L-NAME on sperm capacitation was observed in spermatozoa incubated in BWW-NES (Supplementary Figure S4).

NO^●^ levels were then quantified in capacitating spermatozoa incubated with FCSu in the presence and absence of citrate and the ATP-citrate lyase inhibitor SB204990. In spermatozoa incubated with FCSu alone, NO^●^ levels were significantly increased when compared to the non-capacitated control (Figure 8b). In samples supplemented with 10 mM citrate, levels of NO^●^ were increased further. However, when incubated in capacitating conditions with 30 μM SB204990, NO^●^ levels were decreased in the presence and absence of citrate, highlighting the role of ACLY in NO^●^ production during sperm capacitation.

## 4. Discussion

**Figure 9:**
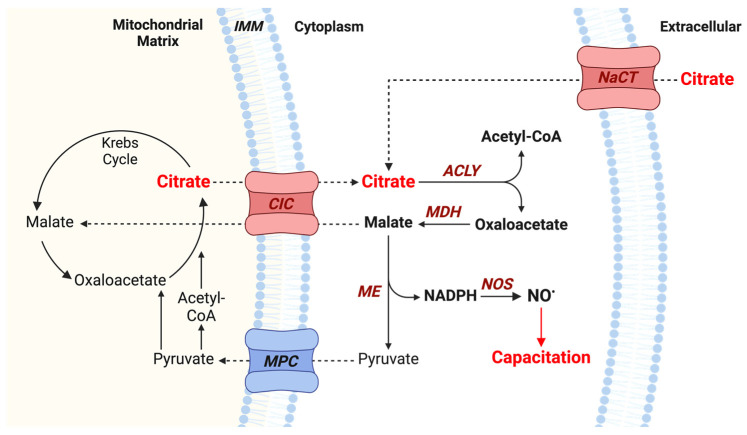
Cytosolic citrate metabolism promotes NO^●^ production in capacitating human spermatozoa. Extracellular citrate can enter the cytosol through carriers such as the sodium-citrate cotransporter (NaCT), and mitochondrial citrate can be transported to the cytosol via the mitochondrial citrate transporter (CIC). Cytosolic citrate is then converted to acetyl-CoA and oxaloacetate via ATP-citrate lyase (ACLY). The oxaloacetate produced is converted by malate dehydrogenase (MDH) to yield malate, which is then converted by the malic enzyme (ME) to NADPH and pyruvate. Pyruvate can re-enter the mitochondrion via the mitochondrial pyruvate carrier (MPC) to continue energy production through the Krebs cycle. NADPH can be used by cytosolic nitric oxide synthase (NOS) to yield nitric oxide (NO^●^) to promote sperm capacitation. Created with BioRender.com (accessed on 8 June 2024).

The results presented in this study reveal a novel role of citrate during human sperm capacitation. Although it is known that spermatozoa are exposed to high concentrations of citrate via seminal plasma, its functions in spermatozoa beyond its canonical role in energy production have remained elusive. In this study, we show evidence that citrate supports sperm capacitation in humans and propose a pathway whereby mitochondrial citrate is exported to the cytosol through the mitochondrial citrate transporter (CIC) and metabolized by ATP-citrate lyase (ACLY) and the malic enzyme (ME) (Figure 9). The NADPH produced in this process can then be used by sperm NOS to produce NO^●^, which is known to modulate tyrosine phosphorylation events in sperm capacitation through activation of cAMP/PKA, PI3K/AKT, and ERK signaling pathways.

Following the incubation of highly motile spermatozoa with FCSu in a medium devoid of energy substrates (BWW-NES), tyrosine phosphorylation levels were higher than that of non-treated cells, indicating that capacitation occurred. This finding highlights that spermatozoa contain sufficient energy metabolites to undergo capacitation, which may originate from the FCSu used or endogenous sources loaded into the cell through contact with the epidydimal fluid and seminal plasma before capacitation. Moreover, the incubation of these samples with 5 and 10 mM citrate resulted in a greater increase in tyrosine phosphorylation at levels similar to spermatozoa incubated with FCSu in BWW-REG containing pyruvate, lactate, and glucose. This finding reveals that, although citrate cannot induce capacitation alone, it supports the capacitation process when the capacitation inducer is present.

When spermatozoa were incubated with high concentrations of citrate (15 and 20 mM), levels of tyrosine phosphorylation in capacitating spermatozoa were decreased at levels similar to the non-capacitated control. This was consistent with decreased total and progressive sperm motility but not sperm viability in the same samples. These findings may result from the dose-dependent increase in intracellular NO^●^ production that was observed after the capacitation media was supplemented with increasing concentrations of citrate. Indeed, although low NO^●^ levels are required for signaling during capacitation, excessive NO^●^ levels would result in oxidative stress that would impair the spermatozoon’s ability to capacitate [43,44,45,46]. Consistent with this study, previous research from our lab has suggested that increasing NO^●^ levels impairs total and progressive sperm motility but not viability [46]. Thus, the spermatozoon must be exposed to an optimal concentration range of citrate for optimal capacitation.

Citrate levels were then quantified in capacitating spermatozoa incubated with FCSu and an inhibitor of the mitochondrial CIC. Unsurprisingly, citrate levels in spermatozoa were significantly higher before capacitation (0 h) than after capacitation (3.5 h), suggesting the utilization of this metabolite by the spermatozoon during capacitation. However, citrate consumption after 3.5 h was significantly lower in spermatozoa treated with FCSu in the presence of the CIC inhibitor compared to that of spermatozoa treated with FCSu alone. This indicates that cytosolic citrate exported from the mitochondrion is metabolized during human sperm capacitation. We also found that 10% FCSu contains about 36 μM citrate, which may partially explain why spermatozoa incubated in BWW-NES with FCSu showed increased tyrosine phosphorylation with no other exogenous sources of energy metabolites present in the capacitation medium.

Capacitating spermatozoa incubated with the mitochondrial CIC inhibitor were then shown to exhibit decreased tyrosine phosphorylation and the inability to undergo the acrosome reaction upon treatment with progesterone, confirming the need of citrate export from mitochondria to support capacitation. This finding agrees with a previous report in which the mitochondrial CIC was shown to play a role in human sperm capacitation [47]. Surprisingly, the supplementation of these samples with 5 mM citrate did not rescue capacitation levels in CIC-inhibited spermatozoa, yet this can be explained by the restriction of CIC-dependent malate import into the mitochondrion, which is used in the citric acid cycle to continue energy production. In addition, the accumulation of cytosolic malate arising from CIC inhibition may result in excessive NO^●^ production based on our proposed pathway, which would promote oxidative stress, thereby impairing capacitation.

The role of ACLY in human sperm capacitation was then investigated. Here, a decrease in tyrosine phosphorylation and acrosome-reacted spermatozoa was observed upon ACLY inhibition in the presence and absence of citrate, demonstrating the requirement of the conversion of citrate to acetyl-CoA and oxaloacetate for sperm capacitation. Consistent with this finding, the incubation of spermatozoa with the capacitation inducer FCSu was shown to increase levels of phosphorylated ACLY (P-ACLY), and the supplementation with citrate, an allosteric activator of the enzyme, led to a further increase in P-ACLY. The increase in ACLY activation corresponded to an increase in tyrosine phosphorylation in the same samples, validating that active ACLY plays a role in the capacitation process.

Following the inhibition of ME with BPA, a similar reduction in tyrosine phosphorylation and acrosome reaction was observed in the presence and absence of citrate. We propose that the NADPH generated by ME downstream of citrate metabolism may be used as a substrate for NOS, providing a mechanism by which citrate metabolism can promote intracellular NO^●^ production during capacitation. However, it should be noted that spermatozoa possess other sources of NADPH. For instance, the activity of glucose-6-phosphate dehydrogenase (G6PD) and 6-phosphogluconate dehydrogenase (6PGD) from the pentose phosphate pathway, as well as cytosolic NADP^+^-dependent isocitrate dehydrogenase (IDPc), can lead to the production of cytosolic NADPH [32,48,49]. While NADPH can originate from different sources, it is important to note that NADPH is used in other cellular processes within the spermatozoon, including the lipid biosynthesis needed for long-term energy storage and the maintenance of peroxiredoxin (PRDX) antioxidant activity, which the spermatozoon requires to protect itself from excessive oxidative damage [31,32,50,51]. Thus, the human spermatozoon is equipped with a sufficient supply of NADPH to support NO^●^ production and PRDX activity to maintain low levels of ROS necessary for sperm capacitation and avoid oxidative stress.

The effects of CIC, ACLY, and ME inhibition on sperm capacitation were not specific to one definite capacitation inducer, since the same inhibitory effects on CIC, ACLY, and ME in FCSu-capacitated spermatozoa were observed in spermatozoa capacitated with the widely used BSA/bicarbonate system. These findings highlight the role of citrate metabolism in the physiological process of sperm capacitation, rather than a specific phenomenon driven solely by FCSu.

Following incubation of capacitating spermatozoa with inhibitors of CIC, ACLY, and ME, we also investigated any potential effects on sperm motility or viability. Importantly, the inhibition of each of these proteins did not affect total motility, progressive motility, or sperm viability, indicating that any decrease in tyrosine phosphorylation was not a result of cell death but rather a direct effect on capacitation.

To determine how citrate may play a role in NO^●^ production, capacitating spermatozoa were incubated with L-NAME, an inhibitor of NOS, in the presence and absence of citrate. Here, it was determined that L-NAME decreased tyrosine phosphorylation in each condition. Our results align well with previous studies in which L-NAME was shown to decrease human sperm capacitation, validating the need for NOS in this process [20,52]. This was observed in spermatozoa incubated in both BWW-REG and BWW-NES, indicating that the effect was not altered by the presence of other exogenous energy substrates. Knowing that ME inhibition results in a similar reduction in sperm capacitation, it can be said that the NADPH generated from ME can be used by NOS in the production of NO^●^ during capacitation. These findings were supported by the decreased intracellular NO^●^ levels observed in capacitating spermatozoa incubated with the ACLY inhibitor SB204990, further reinforcing the relationship between citrate metabolism and NO^●^ production during sperm capacitation.

Altogether, we examined the role of the oxaloacetate generated through cytosolic citrate metabolism. However, it must be noted that the acetyl-CoA generated from ACLY activity may also play an important role in sperm capacitation. For instance, acetyl-CoA may play a role in energy storage in spermatozoa through the initiation of fatty acid synthesis. Fatty acid synthesis may provide the spermatozoon with long-term energy stores that can be used through β-oxidation during the capacitation process, as it has been previously suggested that the energy requirement for spermatozoa to reach the ovum exceeds what is supplied by glycolysis alone [53,54,55]. In addition, the acetyl-CoA produced from cytosolic citrate metabolism may be implicated in protein acetylation, which has been shown to play a role in capacitation. For instance, acetylproteomic studies revealed capacitation-associated functions of lysine-acetylated proteins in human spermatozoa, and inhibition of sperm deacetylases resulted in the onset of phosphorylation of PKA substrates and calcium influx in mouse spermatozoa [56,57,58]. Thus, while this study highlights an important aspect of cytosolic citrate metabolism in the context of ROS generation, further studies would be required to fully understand how citrate supports the process of sperm capacitation.

In this study, the variability of semen samples from different donors must be considered as a potential limitation, since the response to the treatment could vary among individuals. In addition, although we present citrate as a metabolite that supports capacitation, it should be noted that citrate has other effects on the spermatozoon that must be carefully balanced to maintain capacitation. For instance, citrate is a known calcium chelator, and a decrease in intracellular calcium levels may impair the spermatozoon’s motility and ability to capacitate [59,60,61]. However, this potential issue was addressed by adding CaCl_2_ to each BWW medium, allowing the spermatozoa to be exposed to sufficient calcium for capacitation. Citrate is also a known alkalization agent, as its metabolism leads to the production of bicarbonate ions; thus, it can contribute to the increase in intracellular pH and activation of adenylyl cyclase necessary for capacitation [58,59,60].

Understanding the role of citrate metabolism in human sperm capacitation and how this process may be dysregulated in male infertility would greatly help improve current treatment options for infertile couples, such as in vitro fertilization (IVF) or intrauterine insemination (IUI). For instance, pre-treatment of spermatozoa with citrate prior to IVF or IUI, or the inclusion of citrate in commercially available IVF media may simulate the loading of citrate of citrate from the seminal plasma that occurs in the spermatozoa of fertile men. Although further studies are necessary to test the effects of citrate supplementation in these contexts, this presents a potential increase in capacitation in the spermatozoa of infertile men, leading to increased success rates in these assisted reproductive technologies.

## 5. Conclusions

This study reveals a novel role for cytosolic citrate in human sperm capacitation, in a process involving intracellular NO^●^ generation. The incubation of spermatozoa with optimal concentrations of citrate was shown to support capacitation and increase intracellular NO^●^ production, while the presence of inhibitors of mitochondrial CIC, ACLY, ME, and NOS suppressed capacitation. Altogether, these findings provide evidence that citrate exits the mitochondrion through CIC converted by ACLY and ME, yielding NADPH that can be used by NOS to produce intracellular NO^●^ during human sperm capacitation. This research will help understand the regulation of citrate in sperm capacitation, in an effort to improve the overall success rates of current treatment options for infertile couples.

## Data Availability

Data are contained within the article.

## Supplementary Materials

The following supporting information can be downloaded at: https://www.mdpi.com/article/10.3390/antiox13080885/s1, Figure S1: Inhibition of CIC, ACLY, and ME decreases sperm capacitation in BWW-NES; Figure S2: Inhibition of CIC, ACLY, and ME decreases BSA-bicarbonate-induced sperm capacitation; Figure S3: ATP-citrate lyase is activated during sperm capacitation in BWW-NES; Figure S4: Inhibition of NOS decreases sperm capacitation in BWW-NES.
