# Supplementary material for: Citrate Promotes Nitric Oxide Production during Human Sperm Capacitation
Source: Antioxidants (Basel). 2024 Jul 23;13(8):885. doi: 10.3390/antiox13080885 (PMC11352016; doi:10.3390/antiox13080885)
Supplement: Supplementary file 1 [file antioxidants-13-00885-s001.zip › antioxidants-3037138-supplementary.pdf]

Citrate Promotes Nitric Oxide Production during Human Sperm Capacitation

Diego Loggia [1,2,3,4] and Cristian O'Flaherty [1,2,3,4,*]

[1] Department of Pharmacology and Therapeutics, Faculty of Medicine and Health Sciences, McGill University, Montreal, QC H3G 1Y6, Canada
[2] Department of Surgery, Urology Division, Faculty of Medicine and Health Sciences, McGill University, Montreal, QC H4A 3J1, Canada
[3] The Research Institute, McGill University Health Centre, Montreal, QC H4A 3J1, Canada
[4] Department of Anatomy and Cell Biology, Faculty of Medicine and Health Sciences, McGill University, Montreal, QC H3A 0C7, Canada
* Correspondence: cristian.oflaherty@mcgill.ca; Tel.: +1-514-934-1934 (ext. 35410)

Supplemental Figures S1-S4

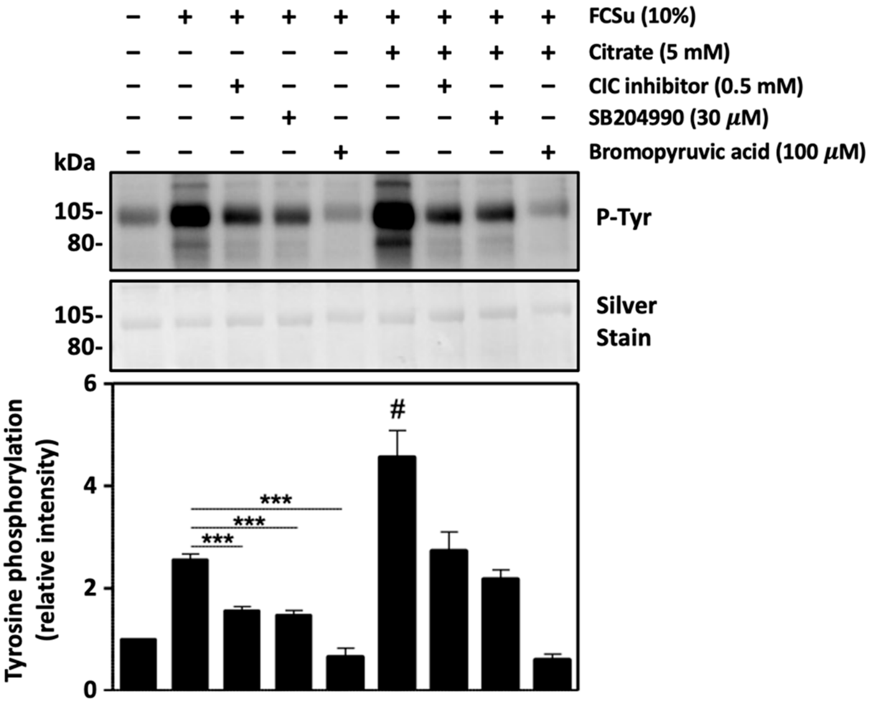

Figure S1. Inhibition of CIC, ACLY, and ME decreases sperm capacitation in BWW-NES. Human spermatozoa incubated in BWW medium with no energy substrates (BWW-NES) show decreased capacitation when incubated with inhibitors of the mitochondrial citrate transporter (CIC inhibitor), ATP-citrate lyase (SB204990) and the malic enzyme (bromopyruvic acid) in the presence and absence of 5 mM citrate, with FCSu as an inducer of capacitation. Relative intensities are expressed as mean ± SEM (# means higher than all other groups, *** $p \leq 0.005$, ANOVA and Tukey's test, $n = 4$).

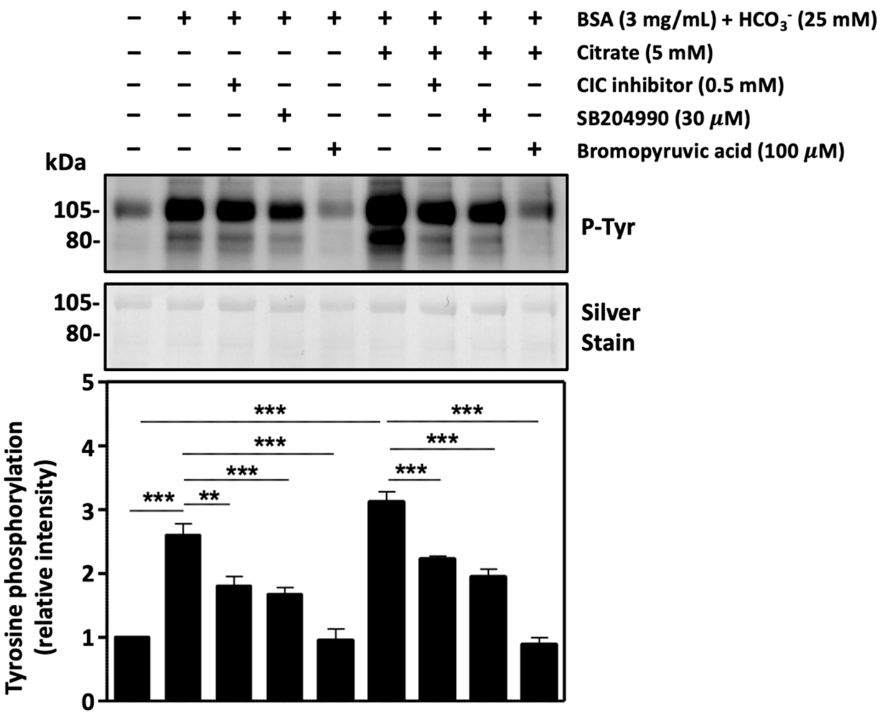

Figure S2. Inhibition of CIC, ACLY, and ME decreases BSA-bicarbonate-induced sperm capacitation. Human spermatozoa incubated in regular BWW medium (BWW-REG) show decreased capacitation when incubated with inhibitors of the mitochondrial citrate transporter (CIC inhibitor), ATP-citrate lyase (SB204990) and the malic enzyme (bromopyruvic acid) in the presence and absence of 5 mM citrate, with BSA and bicarbonate as an inducer of capacitation. Relative intensities are expressed as mean ± SEM (**$p \leq 0.01$, *** $p \leq 0.005$, ANOVA and Tukey's test, $n = 4$).

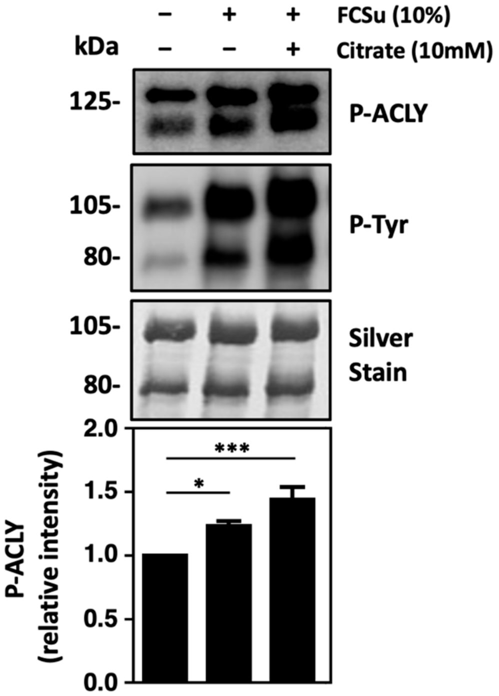

Figure S3. ATP-citrate lyase is activated during sperm capacitation in BWW-NES. Human spermatozoa incubated in BWW medium with no energy substrates (BWW-NES) show increased phosphorylation of ATP-citrate lyase when incubated with FCSu, and a further increase when supplemented with 10 mM citrate. Increased levels of P-ACLY are consistent with increased tyrosine phosphorylation and capacitation in the same samples. Relative intensities are expressed as mean ± SEM (* $p \leq 0.05$, *** $p \leq 0.005$, ANOVA and Tukey's test, $n = 6$).

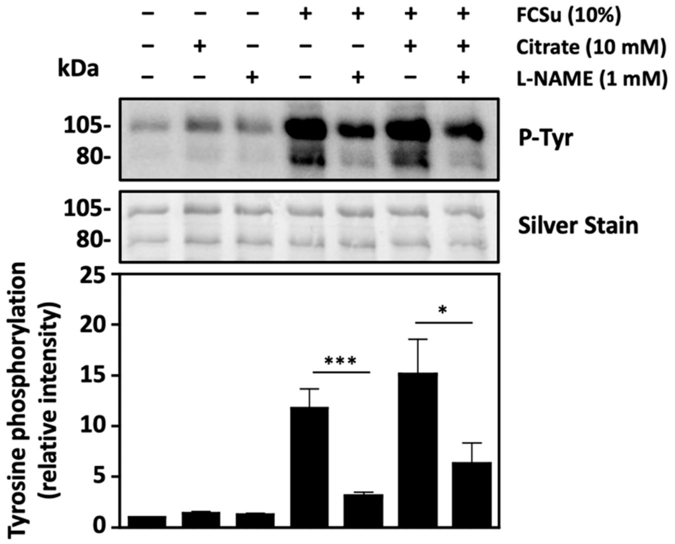

Figure S4. Inhibition of NOS decreases sperm capacitation in BWW-NES. Human spermatozoa incubated in BWW medium with no energy substrates (BWW-NES) show decreased capacitation when incubated with an inhibitors of nitric oxide synthase (L-NAME) in the presence and absence of 10 mM citrate, with FCSu as an inducer of capacitation. Relative intensities are expressed as mean ± SEM (* $p \leq 0.05$, *** $p \leq 0.005$, ANOVA and Tukey's test, $n = 4$).
